# Characterization of the complete chloroplast genome of Jilin ginseng (*Panax ginseng* C. A. Meyer) using next generation sequencing

**DOI:** 10.1080/23802359.2018.1462122

**Published:** 2018-06-18

**Authors:** Kangyu Wang, Li Li, Mingzhu Zhao, Shaokun Li, Honghua Sun, Meiping Zhang, Yi Wang

**Affiliations:** a College of Life Science, Jilin Agricultural University, Changchun, China;; b Research Center for Ginseng Genetic Resources Development and Utilization, Changchun, Jilin Province, China

**Keywords:** Jilin ginseng, *Panax ginseng*, chloroplast genome, HomBlocks, phylogenetic relationship

## Abstract

Jilin ginseng, *Panax ginseng* C. A. Meyer, belongs to the Araliaceae family. It is known as the number one medicinal herb and one of the three native treasures in Northern China and has been cultivated in China for over 2000 years. Jilin ginseng is the staple ginseng in China, which contributes to 85% of the ginseng production in the country, thus becoming one of the most important, rapidly increasing industries in both the Province of Jilin and China. In this study, the complete chloroplast genome of *Panax ginseng* C. A. Meyer was determined by the next generation sequencing. The complete chloroplast genome of Jilin ginseng (*P. ginseng* C. A. Meyer) was 156,286 bp in length and displays a typical quadripartite structure of the large (LSC, 87,127 bp) and small (SSC, 18,329 bp) single-copy regions, separated by a pair of inverted repeat regions (IRs, 25,415 bp each). It harbors 132 functional genes, including 132 protein-coding genes, 37 transfer RNA, and 8 ribosomal RNA genes species. The overall nucleotide composition was: 30.6% A, 31.3% T, 19.4% C, and 18.7% G, with a total G + C content of 38.1%. Phylogenetic relationship analysis shows that *P. ginseng* closely related to *Panax quinquefolius L.*

Jilin ginseng, *Panax ginseng* C. A. Meyer, traditionally known in East Asia, particularly in China, Korea, and Japan, as a medicinal herb (Yun 2001). It is a perennial of the *Araliaceae* family and has been cultivated in China for over 2000 years. Chinese ginseng is mainly grown in Jilin Province, China, and known as Jilin ginseng, and estimated to produce 85% and 70% of the ginseng of China and the world. Modern pharmacological research has focused on the ginsenosides, the major bioactive compound for human health, such as recovery and promotion of vitality, improvement of immune and metabolism systems, regulation of central nervous system, etc (Lee et al. 2008; Su et al. 2018). In this study, we used the next generation sequencing to get the complete chloroplast genome of ginseng, in order to provide information for the study of the origin of medicinal plants in evolution.

The specimen of *P. ginseng* was isolated from Jilin Agricultural University (Jilin ginseng test field in Changchun, Jilin, China) (125.40E; 43.82N) and the DNA of *P. ginseng* was stored in Jilin Agricultural University College of Life Science (No. JLAUCLS5). The Jilin ginseng DNA sample was sequenced using next generation sequencing (Illumina Hiseq4000, CA, USA). The *P. ginseng* complete chloroplast genome was preliminarily annotated using the DOGMA (Dual Organellar GenoMe Annotator) online program (Wyman et al. 2004), with default settings to identify protein-coding genes, rRNAs and tRNAs based on the Plant Plastid Code and BLAST homology searches. The secondary structures of transfer RNA (tRNA) genes were identified by using the ARAGORN (Laslett and Canback 2004) or through manually visual inspection.

The chloroplast genome sequence of *P. ginseng* is a closed-circular molecule of 156,286 bp in length, which is almost the same as the *Panax quinquefoli* chloroplast (156,359 bp). It displayed 132 functional genes set, which is observed in this plant chloroplast, including 87 PCGs, 37 tRNA genes (one for each amino acid, two each for Valine, Asparagine, Serine and Threonine, three each for Leucine and Arginine, three each for Methionine), and 8 genes for ribosomal RNA subunits (two each for *rrn*16, *rrn*23, *rrn*4.5, and *rrn*5). The chloroplast organization of *P. ginseng* is compact, encoding 26,108 bp functional regions (including D-loop region). The annotated chloroplast genome was submitted to GenBank database under accession No.MH049735.

We selected other 20 related complete chloroplast genomes from GenBank to assess the phylogenetic relationship between them. The genome-wide alignment of all plant complete chloroplast genomes was done by HomBlocks (Bi et al. 2017). The phylogenetic trees were reconstructed using maximum likelihood (ML) and neighbour-joining (NJ) methods. ML analysis were performed using RaxML-8.2.4 (Stamatakis 2014), of which the bootstrap values were calculated using 1000 replicates to assess node support. NJ phylogenetic tree was constructed using MEGA7 with 1000 bootstrap replicate (Kumar et al. 2016). All the nodes were inferred with strong support by the ML and NJ methods. As shown in the phylogenetic tree (Figure 1), *P. ginseng* showed the closest with *P. quinquefolius L.*

**Figure 1.**
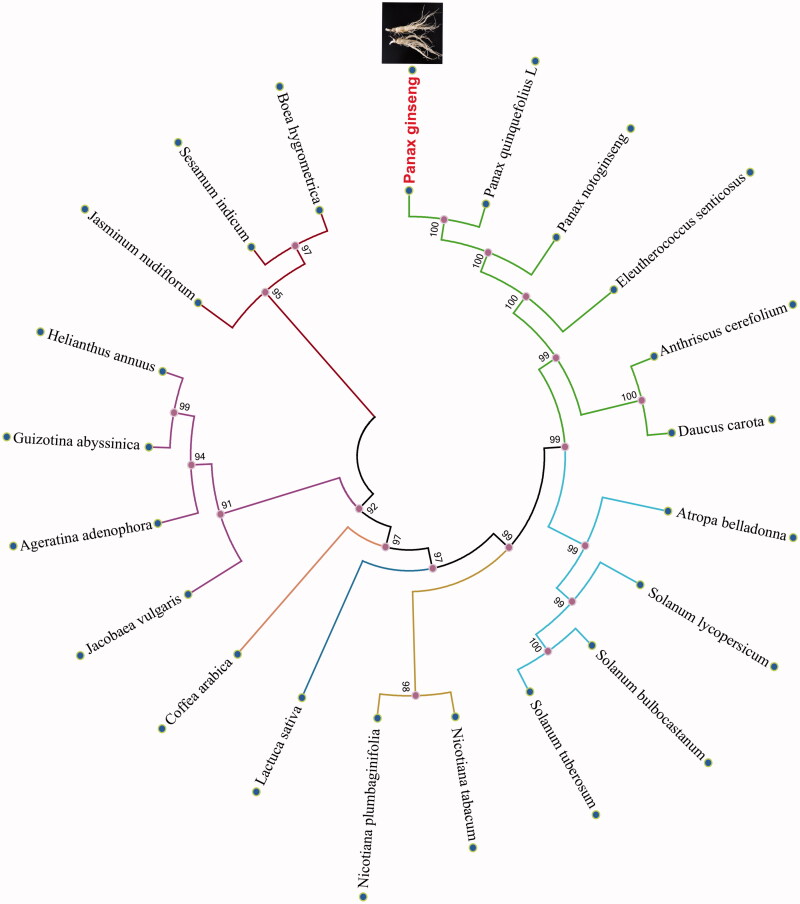
Phylogenetic relationships among 21 plant complete chloroplast genomes. The phylogenetic tree was drawn without setting of an outgroup. All nodes exhibit above 90% bootstraps. The length of branch represents the divergence distance.
